# Stabilization of mouse haploid embryonic stem cells with combined kinase and signal modulation

**DOI:** 10.1038/s41598-017-13471-4

**Published:** 2017-10-16

**Authors:** Haisen Li, Ao Guo, Zhenfei Xie, Wanzhi Tu, Jiali Yu, Huihan Wang, Jian Zhao, Cuiqing Zhong, Jiuhong Kang, Jinsong Li, Shichao Huang, Li Shen

**Affiliations:** 1 State Key Laboratory of Cell Biology, CAS Center for Excellence in Molecular Cell Science, Shanghai Institute of Biochemistry and Cell biology, Chinese Academy of Sciences, Shanghai, 200031 China; 2 Shanghai Key Laboratory of Signaling and Disease Research, Laboratory of Receptor-based Bio-medicine, School of Life Sciences and Technology, Tongji University, Shanghai, 200092 China; 3 Life Sciences Institute, Zhejiang University, Hangzhou, 310000 China; 4 School of Life Science and Technology, ShanghaiTech University, Shanghai, 201210 China; 5 Clinical and Translational Research Center of Shanghai First Maternity and Infant Hospital, Shanghai Key Laboratory of Signaling and Disease Research, School of Life Science and Technology, Tongji University, Shanghai, 200092 China; 6 Laboratory of Muscle Stem Cells and Gene Regulation, National Institute of Arthritis, and Musculoskeletal and Skin Diseases (NIAMS), National Institutes of Health, Bethesda, Maryland 20892 USA

## Abstract

Mammalian haploid embryonic stem cells (haESCs) provide new possibilities for large-scale genetic screens because they bear only one copy of each chromosome. However, haESCs are prone to spontaneous diploidization through unknown mechanisms. Here, we report that a small molecule combination could restrain mouse haESCs from diploidization by impeding exit from naïve pluripotency and by shortening the S-G2/M phases. Combined with 2i and PD166285, our chemical cocktail could maintain haESCs in the haploid state for at least five weeks without fluorescence-activated cell sorting (FACS) enrichment of haploid cells. Taken together, we established an effective chemical approach for long-term maintenance of haESCs, and highlighted that proper cell cycle progression was critical for the maintenance of haploid state.

## Introduction

Mammalian haESCs were first obtained from mouse parthenogenetic blastocysts generated by chemical activation of unfertilized eggs^1–3^. Soon after the establishment of parthenogenetic haESCs (PG-haESCs), androgenetic haESCs (AG-haESCs) were derived by injection of a sperm head into enucleated oocytes or by removal of female pronucleus from zygotes^4–6^. So far, haESCs have been established from parthenogenetic or androgenetic embryos in several species, including mouse, rat, monkey and human^4–9^. These haESCs have only one copy of each chromosome, disruption of one allele can produce a loss-of-function phenotype, providing many possibilities for high-throughput genetic screens^1,10–12^. In addition, PG-haESCs are a powerful tool to generate transgenic mice via injection of genetically modified PG-haESCs into blastocysts^3,9,13^, and AG-haESCs can serve as a substitute for sperm and produce transgenic animals via injecting genetically modified AG-haESCs into oocytes^4–6^. Therefore, haESCs hold great promise for many applications, such as high-throughput genetic screens, generating genetically modified animals, and regenerative medicine^14–18^.

Although haESCs have many advantages, they show a tendency of rapid self-diploidization during cell culture^1,3–9^. Thus, FACS enrichment for haploid cells is required periodically for long-term maintenance of haESCs^1,2,5,8^. Endoreduplication, but not cell fusion, has been shown to be the cause of self-diploidization^3^. Interestingly, Wee1 kinase inhibitor, which accelerates G2-phase checkpoint, has been demonstrated to partially stabilize mouse PG-haESCs and maintain their haploid state for 4 weeks without FACS enrichment^19^, suggesting that G2 to M-phase transition may play an important role in the self-diploidization of PG-haESCs. However, whether accelerating G2 to M-phase transition by Wee1 kinase inhibitor can suppress self-diploidization of AG-haESCs is unknown. In addition, the diploidization of PG-haESCs cannot be completely abolished by promoting G2 to M-phase transition alone^19^, indicating that self-diploidization is also regulated by other factors. Therefore, further optimization of the haESC culture condition is needed to better maintain their haploid state, and the underlying mechanisms of self-diploidization remain to be elucidated.

In this study, we found that a chemical cocktail, namely RDF/PD166285/2i, could stabilize haESCs in the haploid state for at least five weeks without FACS purification, and revealed critical roles of naïve-pluripotency maintenance and cell cycle regulation in inhibiting haESC self-diploidization.

## Results

### Both PG- and AG-haESCs exhibited prolonged G2/M phase

**Figure 1:**
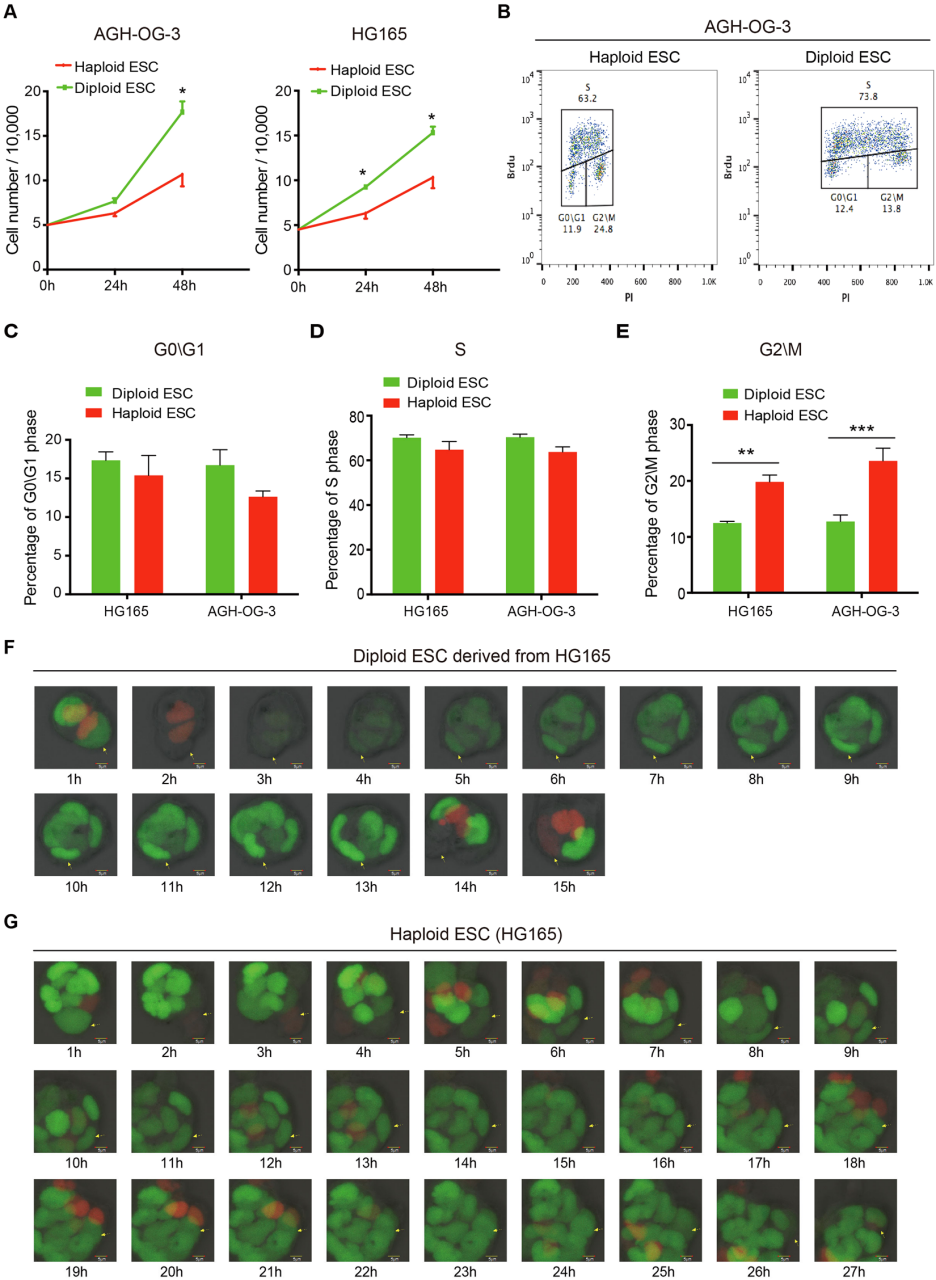
HaESCs show abnormal cell cycle progression. (**A**) Growth rates of haESCs and diploid ESCs derived from AG-haESCs (AGH-OG-3; HG165). Data are shown as means ± sem. *P < 0.05, Haploid ESCs vs diploid ESCs at the same time point. (**B**) Cell cycle analyses of haploid and diploid ESCs derived from AG-haESCs (AGH-OG-3). Haploid (1N) and diploid (4N) cells were isolated simultaneously, and their cell cycle distributions were determined by flow cytometry. (**C**–**E**) Percentages of G0\G1-phase cells (**C**), S-phase cells (**D**), and G2\M-phase cells (E) in haploid and diploid ESCs derived from AG-haESCs (AGH-OG-3; HG165). Data are shown as means ± sem. ^**^P < 0.01, ^***^P < 0.001, Haploid ESCs vs diploid ESCs. (**F** and **G**) Time-lapse imaging of cell cycle-dependent fluorescence changes in diploid ESCs derived from HG165 haESCs (**F**), and in haploid HG165 ESCs (**G**). In this FUCCI system, cells in G1 phase shows orange, and cells in S, G2 and M phases shows green. Arrows indicate tracked cells.

Firstly, we measured the spontaneous diploidization of four different lines of mouse haESCs by FACS analyses. Consistent with the previous reports^1,3,4,6^, the ratio of the haploid G1-phase (1 N) cells in both PG- and AG-haESCs declined gradually over time, whereas the number of diploid G2/M-phase (4 N) cells increased dramatically (Supplementary Fig. S1A). Since abnormal G2 to M-phase transition has been reported to be involved in the self-diploidization of PG-haESCs^19^, we compared the cell cycle profiles between AG-haESCs and the diploid ESCs derived from AG-haESCs to test whether abnormal G2 to M-phase transition also exists in AG-haESCs. Both 1N- and 4N-cells (i.e., haploid and diploid cells, respectively) were sorted out at the same time from two partially diploidized AG-haESC lines (AGH-OG-3 and HG165), and subjected to cell cycle analyses after culturing for a few days. Interestingly, both PG- and AG-haESCs showed a slower proliferation rate compared to the corresponding diploid ESCs (Fig. 1A; Supplementary Fig. S1B), indicating a lengthened cell cycle of the haESCs. Further cell cycle analyses revealed that haESCs consisted of a higher percentage of G2/M-phase cells, and unchanged percentages of G1-phase cells (Fig. 1B–E). To directly visualize cell cycle progression of haploid and diploid ESCs, we employed Fluorescence Ubiquitin Cell Cycle Indicator (FUCCI) technology^20^, and established a HG165-derived AG-haESC line stably expressing Cdt1-tagged-orange and Geminin-tagged-green, where G1-phase and S-G2\M phases were marked by orange and green colors, respectively. We then purified haploid and diploid ESCs from this engineered HG165 ESCs and performed live-cell imaging analyses. Cell cycle progression in diploid ESCs was similar to previous reports^21–25^ (Fig. 1B,F), confirming the successful establishment of the FUCCI reporting system. The FUCCI reporting system also showed significantly longer S-G2\M phases and an unchanged G1-phase duration in haESCs comparing to diploid ESCs (Fig. 1F,G), which was consistent with our FACS-based cell cycle analyses (Fig. 1B–E). Taken together, our results suggested that haESCs grew slower than diploid ESCs due to their atypical cell cycle progression in S-G2\M phases.

### RDF inhibits self-diploidization of haESCs

**Figure 2:**
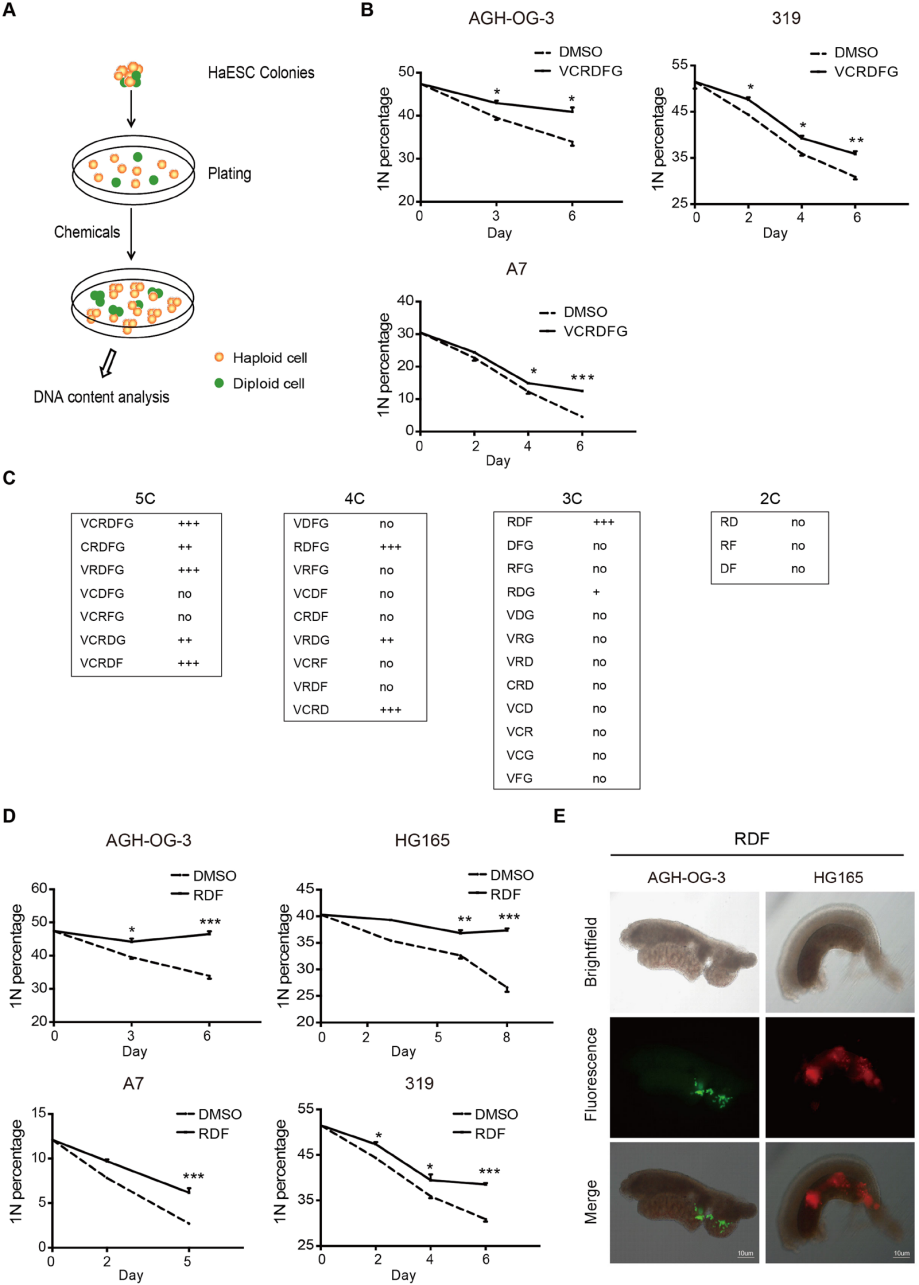
Chemical cocktail RDF restrains the self-diploidization of haESCs. (**A**) Schematic diagram of the screening strategy used to identify chemicals regulating haESC self-diploidization. HaESCs were treated with DMSO or chemical compounds in ES medium, and their DNA contents were analyzed with Hoechst33342 staining. (**B**) Percentage of haploid G1-phase (1N) cells in AG (AGH-OG-3; A7) and PG (319) -haESCs treated with DMSO or VCRDFG (0.5 mM VPA, 3 μM CHIR99021, 1 μM Repsox, 2 μM DMH1, 10 μM Forskolin, 1 μM Compound **E**). DNA contents were determined by flow cytometry every 2 or 3 days. Data are shown as means ± sem. ^*^P < 0.05, ^**^P < 0.01, ^***^P < 0.001, VCRDFG-treated cells vs DMSO-treated cells at the same day. (**C**) Effects of different chemical combinations on self-diploidization. C: chemicals; No: having no influence on haploid stability; ^+^stabilizing haploid ESCs, more plus signs indicate stronger effect on stabilizing haESCs. (**D**) Flow cytometry analyses of 1N cell percentages in AG (AGH-OG-3; HG165; A7) and PG (319) -haESCs treated with DMSO or RDF (Repsox, DMH1, Forskolin). Data are shown as means ± sem. ^*^P < 0.05, ^**^P < 0.01, ^***^P < 0.001, RDF-treated cells vs DMSO-treated cells at the same day. (**E**) Representative gonad images of E13.5 chimeric embryos injected with OCT4-EGFP- or RFP-marked haploid cells. Cells were treated with RDF for 6 days. Top: Brightfield; Middle: Fluorescence; Bottom: Merge.

To effectively maintain the haploid state of haESCs, we adopted a chemical screen strategy to identify small molecules that could regulate diploidization of haESCs. We first tested a group of chemicals that are involved in activating or inhibiting certain signaling pathways (Fig. 2A). However, all tested chemicals alone had no effect on the self-diploidization of haESCs (Supplementary Fig. S1C). Then whether chemical combinations could inhibit self-diploidization was investigated. Indeed, a six-chemical combination, referred to as VCRDFG (consisting of V, VPA, HDACs inhibitor; C, CHIR99021, GSK-3 kinases inhibitor; R, Repsox, inhibitor of TGF-β pathways; D, DMH1, inhibitor of BMP4 pathway; F, Forskolin, adenylate cyclase activator; and G, Compound E, γ-secretase inhibitor) significantly inhibited both AG- and PG-haESCs from self-diploidization, as revealed by the higher ratio of 1 N cells in the presence of VCRDFG (Fig. 2B). In order to identify the essential chemicals required for haploidy stabilization, we further examined different combinations of the six chemicals in VCRDFG. One three-chemical combination (Repsox, DMH1 and Forskolin, termed RDF) was the minimum combination that exhibited similar haploidy stabilization effect as VCRDFG (Fig. 2C,D). In contrast, none of the two-chemical combinations from these three chemicals were sufficient to suppress the diploidization of haESCs (Fig. 2C).

To determine whether RDF impairs the developmental potential of haESCs, we injected wild-type blastocysts with RDF-treated haploid cells that were FACS-purified either from a HG165-derived cell line expressing a RFP reporter or from a AGH-OG-3-derived cell line carrying an Oct4-EGFP transgene^6^. Both RDF-treated cells lines contributed to different tissues (Supplementary Fig. S1D), and more importantly germline cells in the gonads of embryonic day 13.5 (E13.5) embryos (Fig. 2E), demonstrating the developmental potential of haESCs was not impaired by RDF treatment. Our findings therefore demonstrated that RDF treatment could effectively suppress the diploidization of haESCs, without compromising their pluripotency.

### RDF alters the expression of pluripotency and cell cycle genes

**Figure 3:**
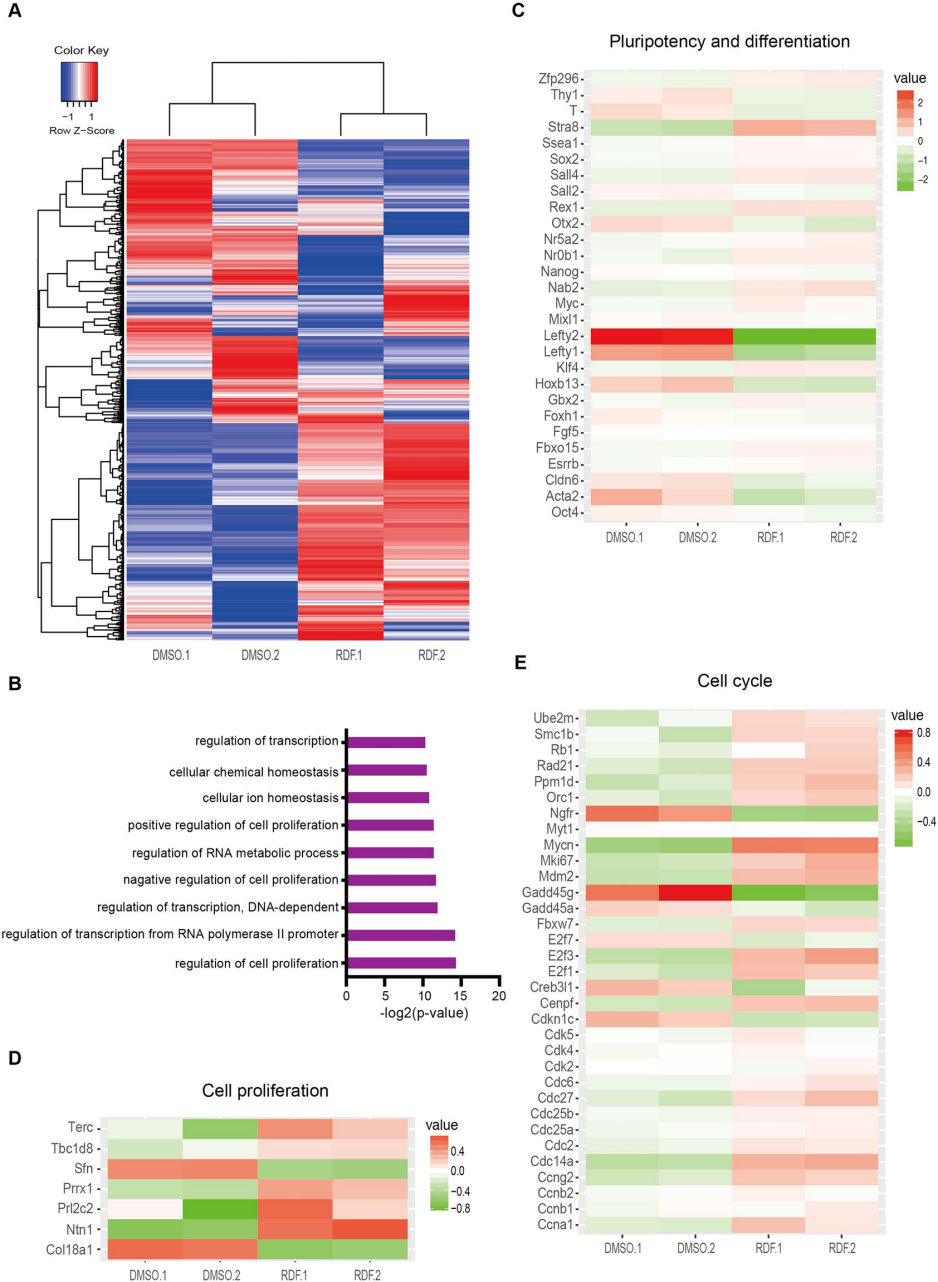
RDF regulates expression profiles of differentiation and proliferation genes. (**A**) Heatmap and hierarchical clustering of RNA-seq data. HaESCs (HG165) treated with DMSO or RDF were compared. (**B**) GO analyses of dysregulated genes (fold change >2). (**C**) Heatmap of pluripotency- and differentiation-related genes in DMSO- and RDF-treated haESCs (HG165). (**D**) Heatmap depicting the expression levels of proliferation genes in haESCs (HG165) treated with DMSO or RDF. (**E**) Heatmap analyses of cell cycle genes in haESCs (HG165) treated with DMSO or RDF.

To explore how RDF suppresses haESC diploidization, we compared the transcriptomes of haESCs treated with RDF and DMSO by using RNA sequencing (RNA-seq) analyses, and identified 475 down-regulated and 440 up-regulated genes (fold change > 2) in the RDF-treated cells (Fig. 3A). Gene ontology (GO) analyses further revealed that the affected genes were significantly enriched in functions relating to proliferation, differentiation and transcription regulation (Fig. 3B). Upon RDF treatment, expression of many naïve pluripotency genes (e.g., *Rex1*, *Ssea1*, *Sall4*, *Zfp296*, *Klf4*, *Nab2*, *Myc*, *Nr5a2*, *Stra8*) were increased, whereas a set of primed pluripotency genes (e.g., *Fgf5*, *T, Lefty1*, *Lefty2*, *Otx2*, *Cldn6*, *Acta2*, *Hoxb13*, *Mixl1*) were down-regulated (Fig. 3C). In addition, genes positively regulating proliferation and cell cycle (e.g., *Terc*, *Ntn1*, *Prrx1*, *Prl2c2*, *Tbcld8*, *Mki67*, *Mycn*, *Cdc14a*, *E2f3*, *Rad21*, *Orc1*, *Rb1*, *Cenpf*) were up-regulated, while genes inhibiting proliferation and cell cycle (*Sfn*, *Col18a1*, *Myt1*, *Gadd45g*, *Creb3l1*, *Gadd45a*, *Cdkn1c*, *Ngfr*) were down-regulated (Fig. 3D,E). Taken together, our data suggested that RDF modulated pluripotency and cell-cycle associated genes.

### RDF reduces the exit from naïve pluripotency

**Figure 4:**
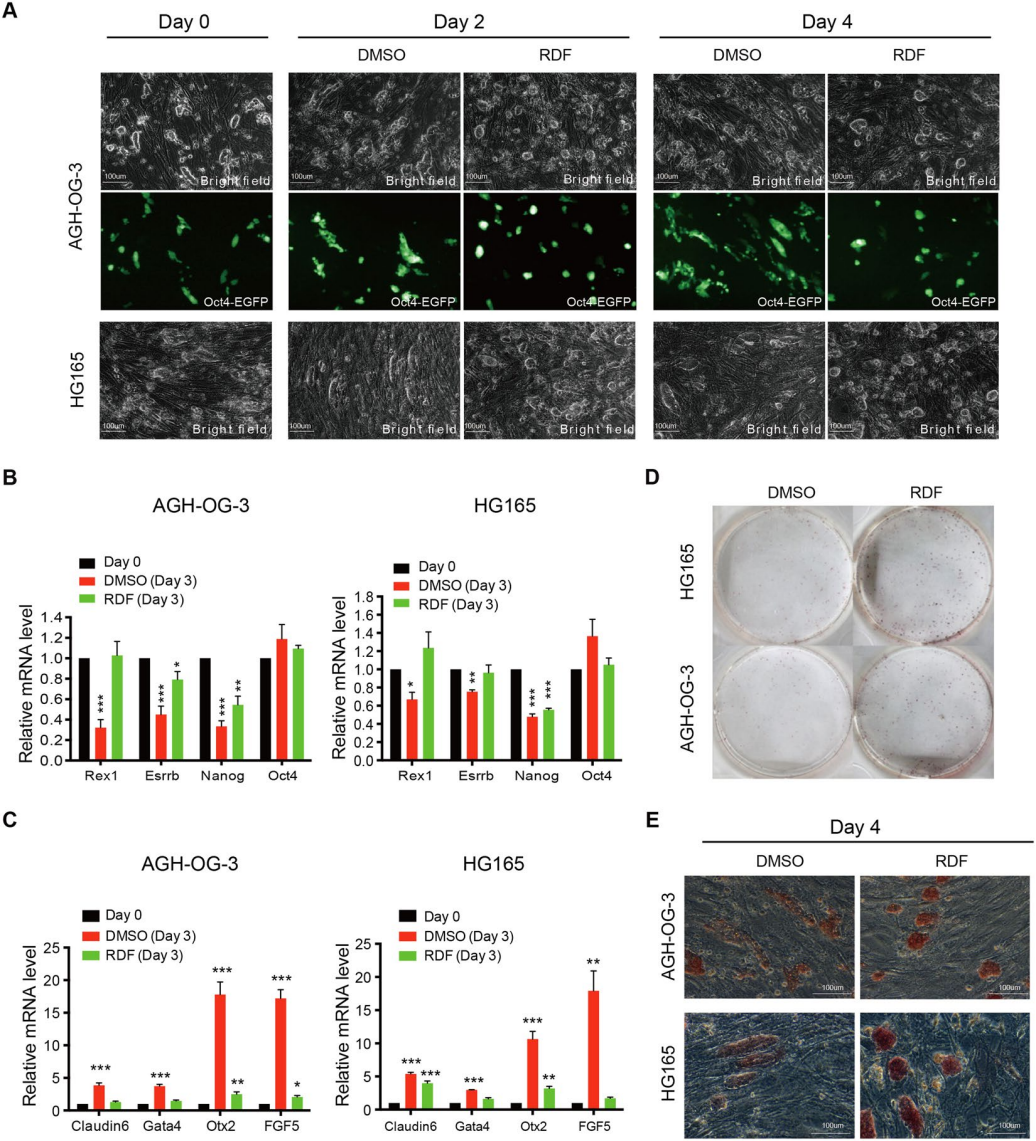
RDF enhances naïve pluripotency of haESCs. (**A**) Representative morphologies of DMSO- and RDF-treated AG-haESCs (AGH-OG-3; HG165). Freshly sorted haESCs were plated on feeder cells, and the images were captured at the indicated time points. Top: phase contrast; Middle: Oct4-EGFP; Bottom: phase contrast. (**B**) Q-PCR analyses of naïve pluripotency markers in AG-haESCs treated as in (**A**). Data are shown as means ± sem. ^*^P < 0.05, ^**^P < 0.01, ^***^P < 0.001, Day 3 cells vs Day 0 cells. (**C**) Q-PCR analyses of primed pluripotency genes in AG-haESCs. Data are shown as means ± sem. ^*^P < 0.05, ^**^P < 0.01, ^***^P < 0.001, Day 3 cells vs Day 0 cells. (**D**) Clonogenicity assay. HaESCs were plated on gelatin-coated plates without feeder cells, and were analyzed for alkaline phosphatase activity. (**E**) Alkaline phosphatase staining of DMSO- and RDF-treated AG-haESCs. HaESCs were cultured in ES medium on feeder cells.

We next assessed RDF’s effect on the pluripotency of haESCs. ESCs have two types of pluripotent states: naïve state and primed state, which can switch from one to the other in different culture conditions^26–30^. Naïve pluripotent ESCs possess tight, dome-shaped colony, low heterogeneity as well as high-level expression of alkaline phosphatase, whereas primed pluripotent ESCs exhibit flat morphology, strong differentiation tendency, high heterogeneity, and low alkaline phosphatase activity^26,29,31–34^. Because a cohort of naïve pluripotency genes was up-regulated and some primed pluripotency genes were down-regulated in RDF-treated haESCs (Fig. 3C), we hypothesized that RDF might impede exit from naïve pluripotency. Indeed, haESCs cultured in the ES medium gradually acquired a flatten morphology, though still expressing Oct4 (Fig. 4A; Supplementary Fig. S2A,E), which is expressed in both naïve and primed pluripotent ESCs. Quantitative real-time PCR (Q-PCR) analyses also showed that naïve pluripotency genes, such as *Rex1*, *Nanog* and *Esrrb* were significantly down-regulated over time (Fig. 4B and Supplementary Fig. S2B), whereas the expression levels of primed pluripotency genes including *Otx2*, *Fgf*5, *Claudin6* and *Gata4* were up-regulated (Fig. 4C and Supplementary Fig. S2C), indicating that haESCs cultured in ES medium tend to gradually exit naïve pluripotency. We found that RDF-treated haESCs maintained dome-shaped colonies morphology (Fig. 4A and Supplementary Fig. S2A), and expressed higher levels of naïve pluripotency markers and lower levels of primed pluripotency markers compared with mock-treated cells (Fig. 4B,C; Supplementary Fig. S2B,C). Consistently, the clonogenic assay showed that RDF treatment notably increased the numbers of alkaline phosphatase-positive colonies (Fig. 4D), and enhanced alkaline phosphatase activity (Fig. 4E and Supplementary Fig. S2D). Immunofluorescence analyses also showed that RDF, as well as the combination of 2i and RDF, promoted the homogeneous expression of Naong in haESCs, which was similar to the effect of 2i (consisting of CHIR99021 and PD0325901) (Supplementary Fig. S2F), suggesting that RDF enhanced the homogeneity of naïve haESCs. Because AG-haESCs were derived in 2i medium^4–6^, we further compared the diploidization rates between AG-haESCs cultured in RDF-supplemented medium and those in 2i-supplemented medium. Interestingly, RDF was more effective than 2i on inhibiting self-diploidization (Supplementary Fig. S2H). Taken together, these results demonstrated that RDF promoted the naïve pluripotency of haESCs and decreased haESC heterogeneity.

### RDF regulates cell cycle transition of haESCs

**Figure 5:**
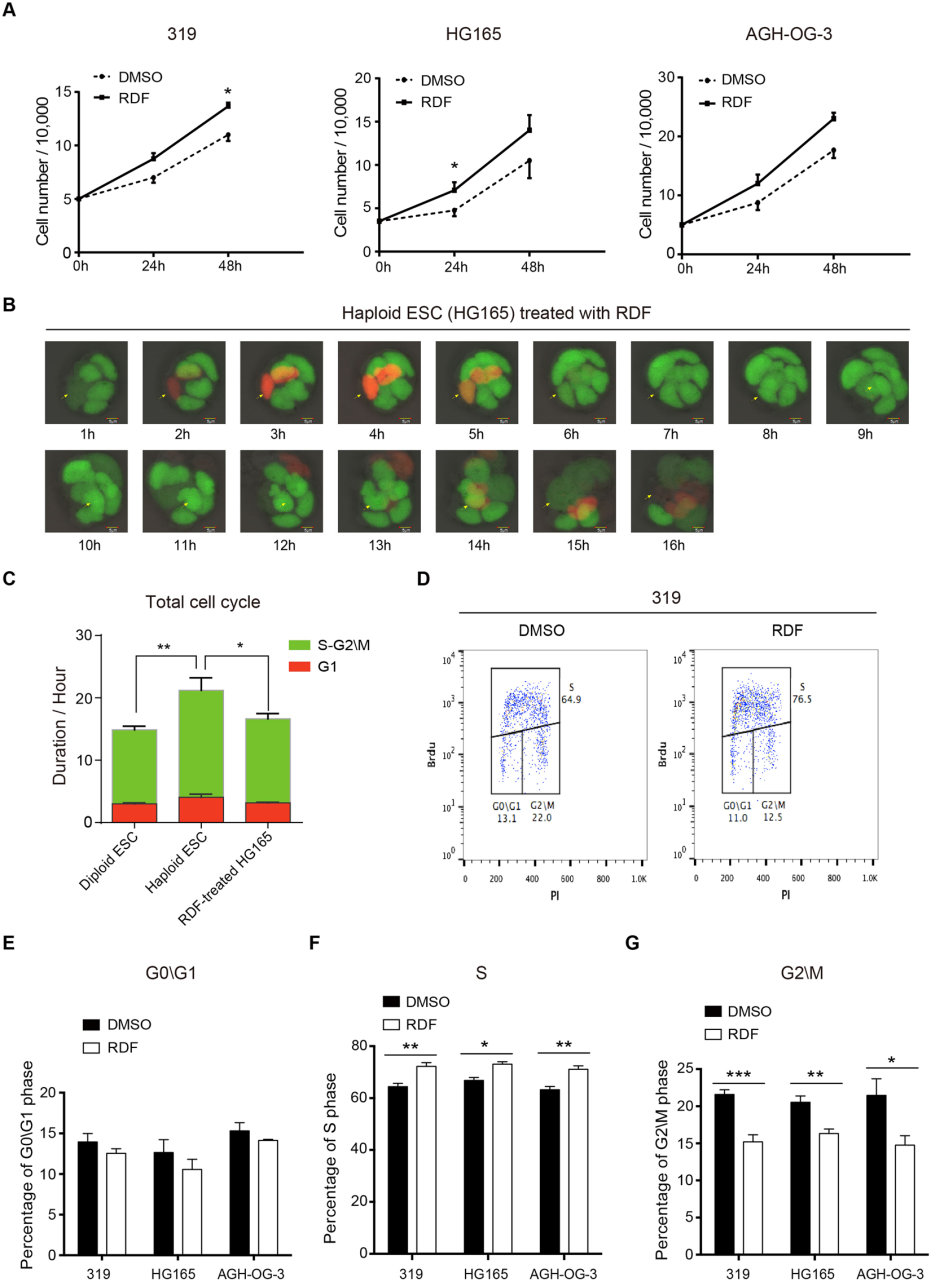
RDF promotes haESC proliferation through accelerating cell cycle progression. (**A**) Growth rates of PG (319)- and AG (HG165; AGH-OG-3)-haESCs treated with DMSO or RDF under feeder-free condition. Data are shown as means ± sem. ^*^P < 0.05, RDF-treated cells vs DMSO-treated cells at the same time point. (**B**) Time-lapse imaging of cell cycle-dependent fluorescence changes in RDF-treated AG-haESCs (HG165). Arrows indicate cells that were tracked. (**C**) Quantification of total cell cycle duration of diploid ESCs, haESCs, and RDF-treated haESCs. The time-lapse images of these cells were shown in Fig. 1F, Fig. 1G, and Fig. 5B, respectively. The haESCs used were the HG165 line, and the diploid ESCs were derived from HG165 haESCs. Data are shown as means ± sem. ^*^P < 0.05, ^**^P < 0.01, Diploid ESC or RDF-treated HG165 vs Haploid ESC. (**D**) Cell cycle distributions in PG-haESCs (319) treated with DMSO or RDF. Freshly sorted haESCs were cultured in ES medium with or without RDF, and their cell cycle distributions were determined by flow cytometry at day 5. (**E**–**G**) The percentages of G0\G1-phase cells (**E**), S-phase cells (**F**), and G2\M-phase cells (**G**) in PG (319)- and AG (HG165; AGH-OG-3)-haESCs treated as in (**D**). Data are shown as means ± sem. *P < 0.05, **P < 0.01, ***P < 0.001, RDF-treated cells vs DMSO-treated cells.

Previous studies have shown that the progression of cell cycle plays an important role in the exit from naïve pluripotent state of ESCs^23,25,35–37^. Therefore, we attempted to determine whether RDF could modulate the cell cycle profile of haESCs. Consistent with our RNA-seq data (Fig. 3C,D), RDF notably shortened the doubling time of haploid ESCs (Fig. 5A), without affecting the apoptosis of haESCs (Supplementary Fig. S3A,B). Interestingly, only the length of S-G2\M phases was shortened by RDF, while the G1-phase length maintained (Fig. 5B,C). In both AG- and PG-haESCs, RDF treatment significantly increased the percentage of S-phase cells and decreased the percentage of G2/M-phase cells, without influencing the percentage of G1-phase cells (Fig. 5D–G). On the contrary, RDF did not alter the cell cycle of haESC-derived diploid ESCs or normal diploid ESCs (Supplementary Fig. S3C–F). These data demonstrated that RDF facilitated haESC proliferation by accelerating S-G2\M-phase progression.

RNA-seq analyses also showed that RDF regulated the expressions of S-phase genes (*Mki67*, *Ccng2*, *Orc1*, *E2f2*, *Gadd45g*, *Gadd45a*, *Cdc6*, *Rrm2*) and G2/M-phase genes (*Ccna1*, *Rad21*, *Rb1*, *Nek1*, *Cenpf*, *Myt1*) (Fig. 3E). We further carried out Q-PCR analyses to validate the expression change of cell cycle regulators. Indeed, RDF treatment significantly reduced the expression of *Myt1*, a negative regulatory gene of G2/M-phase transition, and increased the expression of *Cdc6*, an essential regulator of DNA replication in S phase (Supplementary Fig. S4A,B,C). Although RDF and 2i had comparable effect on promoting pluripotent gene expressions (Supplementary Fig. S2G), only RDF significantly increased the expression level of *Cdc6* (Supplementary Fig. S4D,E), suggesting that RDF but not 2i might stabilize haESCs through regulating cell cycle progression. Since it has been reported that promoter DNA methylation of key regulatory genes could critically regulate cell cycle progression and ESC pluripotency^38–40^, we next examined whether RDF treatment could change the promoter DNA methylation of differentially expressed cell cycle genes. Among the genes we examined, RDF treatment resulted in a decrease of DNA methylation at the *Cdc6* promoter but an increase of DNA methylation at the *Fgf5* promoter (Supplementary Fig. S4F), consistent with their expression changes (Fig. 4C; Supplementary Figs S2C and S4A–E). Our findings suggested that RDF modulated a set of pluripotency and cell cycle genes through altering their promoter DNA methylation.

### RDF/PD166285/2i supports long-term maintenance of haESC haploidy

To compare the effects of RDF and Wee1 kinase inhibitor on haESC haploidy, we firstly treated haESCs with RDF or Wee1 kinase inhibitor (PD166285) in the absence of 2i. PD166285 slightly reduced the diploidization of HG165 (AG-haESC) line (Supplementary Fig. S5A), but not AGH-OG-3 (AG-haESC) and 319 (PG-haESC) lines (Supplementary Fig. S5D,G), suggesting that the inhibitory effect of PD166285 on haESC diploidization was cell line dependent. On the contrary, RDF inhibited the self-diploidization of both AG- and PG-haESCs remarkably (Supplementary Fig. S5A,D and G). Moreover, Q-PCR and western blot analyses revealed that RDF neither changed Wee1 kinase expression at mRNA or protein level (Supplementary Fig. S5B,E,H and J), nor altered the expression of Wee1 kinase upstream regulators (Supplementary Fig. S5C,F and I), indicating that Wee1 kinase inhibitor and RDF functioned through different pathways.

**Figure 6:**
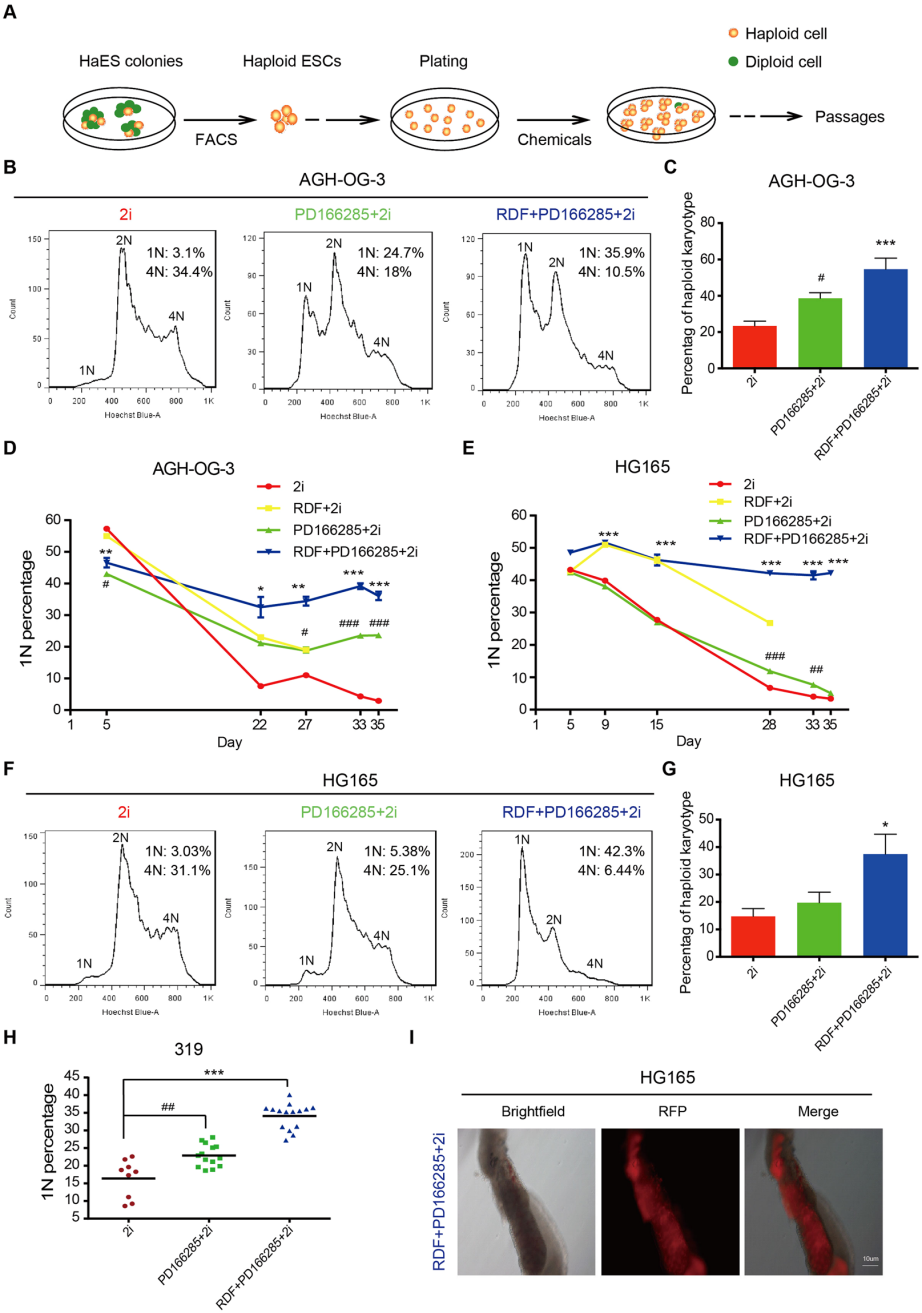
RDF/PD166285/2i supports long-term haploidy maintenance of haESCs. (**A**) Schematic of the experimental design to test the effect of chemicals. (**B**) Representative DNA contents in AG-haESCs (AGH-OG-3) treated with DMSO, 300 nM PD166285 alone, or the combination of RDF and PD166285 in 2i medium for 35 days. Percentages of 1 N and 4N cells were shown. The passage number of AGH-OG-3 was passage 58. (**C**) Percentages of cells with haploid karyotype in AG-haESCs (AGH-OG-3) treated as in (**B**) for 20 days. The number of examined karyotypes in 2i-, PD166285/2i-, and RDF/PD166285/2i-treated cells was 152, 81 and 117, respectively. Data are shown as means ± sem. ^#^P < 0.05, PD166285/2i-treated cells vs 2i-treated cells; ***P < 0.001, RDF/PD166285/2i-treated cells vs 2i-treated cells. (**D**) Flow cytometry analyses of the ratio of haploid 1 N cells in AG-haESCs (AGH-OG-3) treated with DMSO, RDF, PD166285, or the combination of RDF and PD166285 under 2i medium. Data are shown as means ± sem. ^#^P < 0.05, ^###^P < 0.001, PD166285/2i-treated cells vs 2i-treated cells at the same day; ^*^P < 0.05, ^**^P < 0.01, ^***^P < 0.001, RDF/PD166285/2i-treated cells vs 2i-treated cells at the same day. (**E**) Percentages of 1 N cells in AG-haESCs (HG165) treated as in (**D**). Data are shown as means ± sem. ^##^P < 0.01, ^###^P < 0.001, PD166285/2i-treated cells vs 2i-treated cells at the same day; ^***^P < 0.001, RDF/PD166285/2i-treated cells vs 2i-treated cells at the same day. (**F**) Representative flow cytometry results of DNA contents in AG-haESCs (HG165) treated as in (**B**) for 35 days. The ratio of 1N or 4 N cells was represented. The passage number of HG165 was passage 38. (**G**) Percentages of cells with haploid karyotype in AG-haESCs (HG165) treated as in (**B**) for 20 days. The number of examined 2i-, PD166285/2i-, and RDF/PD166285/2i-treated cells was 113, 67 and 97, respectively. Data are shown as means ± sem. ^*^P < 0.05, RDF/PD166285/2i-treated cells vs 2i-treated cells. (**H**) Single colony assay. Single colonies derived from PG-haESCs (319) were cultured in 2i/LIF medium supplemented with DMSO, PD166285, or the combination of RDF and PD166285 for 35 days. Data are shown as means ± sem. ^##^P < 0.01, PD166285/2i-treated cells vs 2i-treated cells; ***P < 0.001, RDF/PD166285/2i-treated cells vs 2i-treated cells. (**I**) Representative images of the chimeric gonads. RFP fluorescence suggested the chimeric contribution of HG165-haploid cells. Cells were cultured in RDF/PD166285/2i condition for 15 days before injection. Left: Brightfield; Middle: Fluorescence; Right: Merge.

We subsequently examined the effectiveness of RDF in the presence of 2i. Surprisingly, RDF decreased cell proliferation of haESCs in 2i condition (Supplementary Fig. S6A–C), leading to significant cell death after prolonged culture. However, when both RDF and PD166285 were added to the 2i culture medium, the cells could proliferate normally probably due to the growth promotion effect of PD166285 (Supplementary Fig. S6A–C). More importantly, the combination of RDF and PD166285 functioned better than RDF alone in stabilizing haploidy (Supplementary Fig. S6D). Therefore, RDF/PD166285/2i appeared to be an ideal culture condition for haESCs. To further evaluate the effectiveness of RDF/PD166285/2i, 1N cells freshly purified from haESCs were immediately cultured in 2i medium supplied with different chemicals for a relatively long period (Fig. 6A). We found that haESCs treated with RDF/PD166285/2i exhibited a very high ratio of haploid 1N cells and an evidently low ratio of diploid 4 N cells (Fig. 6B and F). Karyotype analyses also showed that haESCs treated with RDF/PD166285/2i displayed a higher percentage of haploid karyotype cells compared with haESCs treated with PD166285/2i or 2i alone (Fig. 6C,G; Supplementary Fig. S6E), consistent with FACS profiles. The haploid state could be well maintained for five weeks after RDF/PD166285/2i treatment without FACS enrichment (Fig. 6D,E). In addition, single colony assay revealed that the survived colonies after RDF/PD166285/2i treatment for 35 days contained a significant higher percentage of haploid 1N cells, compared with the survived colonies cultured in 2i medium supplied with or without PD166285 (Fig. 6H). Interestingly, RDF/PD166285/2i not only increased *Cdc6* expression, but also upregulated the expression of *Cdk2*, which is essential in S- and G2-phase (Supplementary Fig. S6F). Because Wee1 kinase has been reported to inhibit Cdk2^41^, RDF/PD166285/2i treatment might up-regulate *Cdk2* through inhibiting Wee1 kinase by PD166285. Importantly, haESCs cultured in RDF/PD166285/2i condition could efficiently contribute to somatic and germline cells in E13.5 chimeric embryos (Fig. 6I and Supplementary Fig. S6G). Thus, our data suggested that the RDF/PD166285/2i combination  was suitable for long-term maintenance of haESCs.

## Discussion

Abnormal cell cycle progression, such as cell cycle arrest, induces ESC differentiation or apoptosis^42,43^. Compared to most other cells, ESCs exhibit a shortened cell cycle, characterized by a short G1-phase and a high proportion of S-phase cells. However, the lengths of G2- and M-phases are comparable between ESCs and other cells^44–46^. Our results showed that haESCs exhibited a prolonged G2/M phase compared to diploid ESCs, and RDF could shorten the duration of S-G2/M phases from 19 hours to 13 hours, suggesting that RDF might inhibit haESC diploidization by reducing the errors in S-G2/M phases and shortening the duration of S-G2/M phases. This is consistent with our recent report that prolonging prometaphase/metaphase of M phase by nocodazole or STLC treatment increased the diploidization rate of haESCs, whereas shortening prometaphase/metaphase by Aurora B overexpression significantly decreased self-diploidization rate^47^.

Environmental cues can trigger different responses in ESC cultures, leading to distinct gene expression networks under different culture conditions^48,49^. In this study, we found that RDF/PD166285 could facilitate long-term maintenance of haESCs in the 2i culture medium, but not in the ES medium, indicating that the culture condition is also critical in stabilizing the haploid state. It has been reported that PD166285 not only targets Wee1 kinase, but also inhibits the tyrosine kinase c-Src. While c-Src is involved in stimulating mouse ESC proliferation in the ES medium^50^, its activation promotes differentiation in the 2i medium^51^. Therefore, by inhibiting c-Src, PD166285 might block RDF’s proliferation promotion effect on haESCs in the ES medium, but functions together with RDF in the 2i culture medium to promote cell proliferation and naïve pluripotency, resulting in an effective stabilization of haESC haploidy.

Self-diploidization is an intrinsic feature of haESCs, and the diploidization rate varies among different cell lines. For example, PG-haESC line 319 showed a higher diploidization rate than two AG-haESC lines (AGH-OG-3 and HG165). And among the AG-haESC lines used in this study, the A7 line which was derived from an old mouse exhibited higher diploidization rate than the other two lines derived from young mice (AGH-OG-3 and HG165), suggesting that the age of mice from which haESCs were derived might also affect self-diploidization rate. Thus, it is important to use multiple independent haESC lines to study the mechanism of self-diploidization. Using multiple haESC lines, we found that both AG- and PG-haploid ESCs exhibited lower proliferation rates than diploid ESCs. Interestingly, such proliferation difference between haploid and diploid ESCs was not observed in previous reports^1,7^, therefore examining the haESC lines used in those studies might provide further insight into the mechanism of haESC self-diploidization.

ESC differentiation provides a competitive strategy for tissue regeneration and cell therapy^52,53^. Recently, it has been reported that mouse ESCs could generate functional haploid gametes (i.e., haploid spermatid-like cells and female mature oocytes) *in vitro* ^54,55^. However, such *in vitro* gametogenesis of diploid ESCs required gonadal somatic cells derived from embryos, limiting the application of this approach in human clinical research. Given the haploid nature of haESCs, it is highly possible that under appropriate conditions haESCs could differentiate into haploid gametes efficiently without the need of embryo-derived gonadal somatic cells. Despite their unstable nature, haESCs also provide new possibilities in many aspects, including facilitating large-scale genetic screen and understanding genome evolution and functions. Thus, our study not only reports an effective chemical cocktail that supports long-term maintenance of haESC haploidy, but also promotes future applications of haESCs in many exciting research areas.

## Methods

### Derivation of haESCs

All animal methods were performed in accordance with the guidelines of the Institute of Biochemistry and Cell Biology, Chinese Academy of Sciences. All experimental procedures were authorized by the Animal Care and Use Committee of the Shanghai Institute of Biochemistry and Cell Biology, Chinese Academy of Sciences. Mouse haESCs were established as previously described^1,2,4,6^. All AG- and PG-haESCs used in this study were established from C57BL/6 mice. Androgenetic A7-haploid cell line was derived from elderly mouse, while androgenetic haploid cell lines AGH-OG-3 and HG165 were generated from young mice. Androgenetic AGH-OG-3 and parthenogenetic 319 haESCs were established from OCT4-EGFP transgene mice. For chimera formation experiments, HG165 haESCs were transfected with a piggyBac transposon vector to stably express RFP.

### HaESCs culture

Mouse AG- and PG-haESCs were cultured on feeder cells with ES medium or 2i culture medium^54^. ES medium consisted of DMEM with 15% fetal bovine serum (Thermo Fisher Scientific), 1% nucleosides (EMD Millipore), 1% glutamax (Thermo Fisher Scientific), 1% non-essential amino acid (Thermo Fisher Scientific), 1% β-mercaptoethanol (EMD Millipore), 1000 U/ml leukemia inhibitory factor (LIF; EMD Millipore), 100 U/ml penicillin and 100 μg/ml streptomycin. 2i culture medium contained the above ES medium supplemented with 3 μM CHIR99021 (Tocris) and 1 μM PD0325901 (Tocris). In general, haESCs were cultured in ES medium, unless noted otherwise. Feeder cells were not used in most of our experiments except in haploid percentage analyses, morphology analyses, and alkaline phosphatase staining. For single colony assay, 16 single colonies were segregated from PG-haESCs (the 319 line), and sequentially passaged in ES medium supplemented with 2i, PD166285/2i, or RDF/PD166285/2i.

### Flow cytometry analysis and cell sorting

HaESCs were trypsinized, and stained with Hoechst33342 for 30 mins at 37 °C. For cell sorting, haploid G1-phase and diploid G2\M-phase cells were purified on BD FACSAria^TM^ II machine. For flow cytometry, the DNA contents of haESCs with or without chemicals treatment were detected on BD LSR II  machine. Cell apoptosis assay was carried out following the manufacturer’s protocol (Biolegend, 640930). Data were analyzed with the FlowJo software (Tree Star).

### Real-time quantitative PCR

Total RNA was isolated from haESCs with TRIzol (Sigma) reagent, and then reverse transcribed with M-MLV Reverse Transcriptase (Promega) following manufacturers’ instructions. Real-time quantitative PCR was performed on Stratagene MX3000P (Agilent Technologies) with 2 × JumpStart TaqReadyMix (Sigma) and EvaGreen Dye (Biotium). The relative expression values were normalized to the internal control *Gapdh*. Primer sequences are listed in Table 1.

**Table 1:** RT-qPCR Primer Sequences.

Gene	Sense (5′ to 3′)	Anti-sense (5′ to 3′)
Rex1	TCCAAGGAGCTGAACTCCT	CGTCTTGCTTTAGGGTCAGTT
Nanog	CAGGTGTTTGAGGGTAGCTC	CGGTTCATCATGGTACAGTC
Esrrb	CATGAAATGCCTCAAAGTGGG	AAATCGGCAGGTTCAGGTAG
Oct4	TCTTTCCACCAGGCCCCCGGCTC	TGCGGGCGGACATGGGGA GATCC
Otx2	CCATGACCTATACTCAGGCTTCAGG	GAAGCTCCATATCCCTGGGTGGAAAG
Claudin6	TGCAAGGTGTATGACTCACTGT	GACGAGACTTGGAGTTCCTATCT
Fgf5	AAAGTCAATGGCTCCCACGAA	GGCACTTGCATGGAGTTTTCC
Gata4	CCTGGAAGACACCCCAATCTC	AGGTAGTGTCCCGTCCCATCT
Cdk2	CCTGCTTATCAATGCAGAGGG	TGCGGGTCACCATTTCAGC
Cdk4	ATGGCTGCCACTCGATATGAA	TCCTCCATTAGGAACTCTCACAC
Cdk6	GGCGTACCCACAGAAACCATA	AGGTAAGGGCCATCTGAAAACT
Cyclin D	GCGTACCCTGACACCAATCTC	CTCCTCTTCGCACTTCTGCTC
Cyclin E1	GTGGCTCCGACCTTTCAGTC	CACAGTCTTGTCAATCTTGGCA
Cyclin A	GCCTTCACCATTCATGTGGAT	TTGCTCCGGGTAAAGAGACAG
Cdc2	CGAGTTCACACGACAAGCCT	GGAGTCACAAACGGTTCAGTTC
Cyclin B	AAGGTGCCTGTGTGTGAACC	GTCAGCCCCATCATCTGCG
Cdk5	CCCTGAGATTGTGAAGTCATTCC	CCAATTTCAACTCCCCATTCCT
Myt1	ACCTCCCATACCTCTGTCCAG	GCGAACCTCCACGATGACTG
Cdc25a	TTGCGGGCTGTTTGACTCC	GGGTCACTGTCCAAAATGTTCT
Cdc25b	TCCGATCCTTACCAGTGAGG	GGTCTCTGGAAGCGCACATT
Cdc25c	GTTTCAGCACCCAGTTTTAGGT	AGAATGCTTAGGTTTGCCGAG
Cdc6	TGGCATCATACAAGTTTGTGTGG	CAGGCTGGACGTTTCTAAGTTTT
E2f1	CTCGACTCCTCGCAGATCG	GATCCAGCCTCCGTTTCACC
Fzr1	GTTTCAGAGATGCGGAGAACC	CAGGCCGTCTTTGCCATTG
Wee1 kinase	GTCGCCCGTCAAATCACCTT	GAGCCGGAATCAATAACTCGC
Fgfr1	GCAGAGCATCAACTGGCTG	GGTCACGCAAGCGTAGAGG
Cry1	CACTGGTTCCGAAAGGGACTC	CTGAAGCAAAAATCGCCACCT
Cry2	CACTGGTTCCGCAAAGGACTA	CCACGGGTCGAGGATGTAGA
Bmal1	TTTGTTTGTCGTAGGATGTGACC	CGCAGTGTCCGAGGAAGATA
Gapdh	AGGTCGGTGTGAACGGATTTG	TGTAGACCATGTAGTTGAGGTCA

### Clonogenicity assay and alkaline phosphatase staining

For clonogenicity assay, 600 haESCs were plated in one well of a 6-well plate coated with gelatin, and cultured in ES medium added with DMSO or RDF for 3 days. These cells were fixed with 4% paraformaldehyde solution and then stained for alkaline phosphatase activity. The procedures of alkaline phosphatase staining were performed as described previously^56^. Alkaline phosphatase-positive colonies were captured under Zeiss fluorescence microscope.

### Cell cycle percentage analyses

To avoid the contamination of haploid cell-derived diploid cells in haESC cultures, freshly sorted haESCs were used within seven days. ESCs were labeled with 10 μM BrdU for 30 mins at 37 °C, and fixed with 70% ethanol overnight at 4 °C. After treated with 2N HCl/0.5% Triton X-100 solution for 30 mins at room temperature, cells were neutralized with 0.1 M sodium borate for 2 mins. Cells were incubated with anti-Brdu antibody (1:500, AbD Serotec) for 1 hour at room temperature, and stained with Alexa Fluor 488 secondary antibody (1:500) for 30 mins. Then, cells were treated with 10 μg/ml RNase A and 20 μg/ml propidium iodide for 30 mins. The stained cells were analyzed on BD LSR II machine.

### Injection of haESCs into blastocysts

To generate chimeric mice, haESCs were injected into diploid blastocysts as described previously^6^. Pregnant ICR receipts were dissected at E13.5. The successful chimaerism was judged by OCT4-EGFP or RFP expression. Images were collected under Olympus SZX16 microscope (Olympus, Japan).

### FUCCI imaging analyses of haESC cell cycle

The cDNA encoding mKO2-hCdt1 or mAG-hGeminin was amplified from pFucci-G1 Orange or pFucci-S/G2/M Green plasmids (Medical & Biological Laboratories Co, LTD) and cloned into PiggyBac Transposon vectors. The reconstructed PiggyBac transposon vectors containing mKO2-hCdt1 and mAG-hGeminin were transfected into HG165 haESCs. The haploid and diploid ESCs simultaneously expressing both mKO2-hCdt1 and mAG-hGem were sorted, and then cultured in 8-well Chamber Slides (Lab Tek). Live imaging of haploid and diploid ESCs was performed with FV1200MPE Laser Scanning Microscopes (Olympus) under the 60x/1.3 silicon oil UPLSAPO60XS objective lens. Images were taken every 30 mins with z-stacks for more than 24 hours, and analyzed by Olympus FV10-ASW 4.2 software.

### Immunofluorescence staining

HaESCs cultured in Chamber Slides were fixed by 4% paraformaldehyde solution for 15 mins at room temperature. Blocking and permeabilization buffer (1% BSA, 0.5% Triton X-100 in PBS) was used to permeabilize the cells for 1 hour at room temperature. Cells were then incubated overnight with Oct-3/4 (sc-5279; Santa Cruz) and Nanog (sc-33760; Santa Cruz) antibodies at 4 °C, and then incubated with fluorescent conjugated secondary antibodies for 1 hour at room temperature. Nuclei were counterstained with DAPI. Images were captured with Zeiss LSM 710 Confocal Scanning Microscope.

### Metaphase Chromosome Spread Analysis

HaESCs were cultured in 6-well plates for 2 days prior to the preparation of chromosome spreads. Cells were treated with 0.1 μg/ml Colcemid at 37 °C for 2 hours and then trypsinized. Cells were incubated with hypotonic solution (0.56% KCl) for 6 mins and fixed by 3:1 methanol:acetic acid for 10 mins at room temperature. Fixation was repeated three times following centrifugation and resuspension in fixative solution. Meataphase spreads were prepared on slides and stained with DAPI.

### RNA-seq library preparation and data analysis

Total RNA was purified with an RNeasy Plus Mini kit (Qiagen). 150 ng of purified RNA was subjected to mRNA isolation and library preparation with a VAHTS Stranded mRNA-seq Library Prep Kit for Illumina (Vazyme) following manufacturer’s instructions. Libraries were pooled and sequenced on an Illumina HISEQ 2500. RNA-seq data analysis was performed with Tophat and Cufflinks using the UCSC mm9 annotation. The Gene Expression Omnibus accession number for the RNA-seq data generated in this study is GSE104519.

### DNA methylation analysis

Genomic DNA was extracted from haESCs with TIANamp Genomic DNA Kit following manufacturer’s instructions. Promoter CpG methylation of primed pluripotent and cell cycle genes was analyzed as previously described^57^. The sequences of bisulfite PCR primers are provided in Table 2.

**Table 2:** Bisulfite PCR Primer Sequences.

Gene	Forward (5′ to 3′)	Reverse (5′ to 3′)
Fgf5 outside	GGTGTTGGGTAAAGAGTGAGTTGG	AAAAACAAAAACAAACTCATTCTTC
Fgf5 inside	GGTAAAGAGTGAGTTGGTTGGGAT	AATCCTAAATACATCTTACAAAACTAACTC
Cdc6 outside	GGGAGGTTGGGTGGAGGATA	AAACTTATATTAAATAACTTCTTTTTCAA
Cdc6 inside	TGGGTGGAGGACAAAGTAGAAATAA	ATATTAAATAACTTCTTTTTCAA

### Statistical Analysis

Quantitative results were presented as mean ± SEM. One-way ANOVA or Student’s t test was used for multiple- or two-sample-comparisons, respectively.

## Electronic supplementary material

Supplementary information
