# Supplementary material for: Stabilization of mouse haploid embryonic stem cells with combined kinase and signal modulation
Source: Sci Rep. 2017 Oct 16;7:13222. doi: 10.1038/s41598-017-13471-4 (PMC5643530; doi:10.1038/s41598-017-13471-4)

Stabilization of mouse haploid embryonic stem cells with combined kinase and signal modulation

Haisen Li[1, 6, 7], Ao Guo[1, 7], Zhenfei Xie[1, 7], Wanzhi Tu[4], Jiali Yu[3], Huihan Wang[3], Jian Zhao[1], Cuiqing Zhong[1], Jiuhong Kang[5], Jinsong Li[1], Shichao Huang[2, *] and Li Shen[3, *]

[1] State Key Laboratory of Cell Biology, CAS Center for Excellence in Molecular Cell Science, Shanghai Institute of Biochemistry and Cell biology, Chinese Academy of Sciences, Shanghai 200031, China

[2] Shanghai Key Laboratory of Signaling and Disease Research, Laboratory of Receptor-based Bio-medicine, School of Life Sciences and Technology, Tongji University, Shanghai 200092, China

[3] Life Sciences Institute, Zhejiang University, Hangzhou 310000, China

[4] School of Life Science and Technology, ShanghaiTech University, Shanghai 201210, China

[5] Clinical and Translational Research Center of Shanghai First Maternity and Infant Hospital, Shanghai Key Laboratory of Signaling and Disease Research, School of Life Science and Technology, Tongji University, Shanghai 200092, China

[6]Laboratory of Muscle Stem Cells and Gene Regulation, National Institute of Arthritis, and Musculoskeletal and Skin Diseases, National Institutes of Health, Bethesda, Maryland 20829, USA

[7] These authors contributed equally to this work

*Correspondence: li_shen@zju.edu.cn (L.S.), and huangshichao@sibcb.ac.cn (S.H.)

SUPPLEMENTARY FIGURE LEGENDS

Supplementary Figure S1. Mouse haESC diploidization and chemicals screening summary

(A) Flow cytometry analyses of DNA contents in AG- and PG- haESCs. AG-haESCs included cell lines AGH-OG-3, HG165 and A7, while cell line 319 belonged to PG-haESCs. The percentage of 1N or 4N cells was indicated.

(B) Growth rate analyses of haESCs and diploid ESCs derived from PG-haESCs (319). Data are shown as means ± sem. $^{*}P < 0.05$, Haploid ESC vs Diploid ESC at the same time point.

(C) Summary of the influence of single chemical treatment on the haploid stability of haESCs.

(D) Representative images of day 13.5 chimeric embryos formed by injection of RFP-marked haploid cells treated by RDF for 6 days. Left: Brightfield; Middle: Fluorescence; Right: Merge.

Supplementary Figure S2. RDF promotes naïve pluripotency of PG-haESCs and reduces haESC heterogeneity

(A) Representative morphologies of PG-haESCs (319) cultured in ES medium supplemented with DMSO or RDF on feeder cells. The images of ESCs morphology were captured every 2 days. Top: phase contrast; Bottom: Oct4-EGFP.

(B) Q-PCR analysis of naïve pluripotent genes in PG-haESCs treated as in (A). Data are shown as means ± sem. $^{*}P < 0.05$, $^{**}P < 0.01$, $^{***}P < 0.001$, Day 3 cells vs Day 0

cells.

(C) Q-PCR analysis of primed pluripotent genes in PG-haESCs treated as in (B). Data are shown as means ± sem. $^{*}P < 0.05$, $^{***}P < 0.001$, Day 3 cells vs Day 0 cells.

(D) Representative images of AP-staining positive PG-haESCs cultured in ES medium.

(E) Western blot analyses of PG (319) and AG (AGH-OG-3) -haESCs cultured in ES or 2i-ES medium for 3 days. Beta-actin was used as loading control.

(F) Immunofluorescence staining of Nanog and Oct4. AG-haESCs (HG165; AGH-OG-3) were cultured in ES medium, and treated with DMSO, RDF, 2i, or the combination of RDF and 2i.

(G) Q-PCR analysis of naïve pluripotent genes. AG-haESCs (AGH-OG-3) were cultured in ES medium added with DMSO, 2i, or RDF. Data are shown as means ± sem. $^{\#}P < 0.05$, $^{\#\#\#}P < 0.001$, 2i-treated cells vs DMSO-treated cells; $^{*}P < 0.05$, RDF-treated cells vs DMSO-treated cells.

(H) Flow cytometry analyses of the ratio of 1N cells in AG-haESCs (HG165) treated with DMSO, RDF, or 2i in ES medium. Data are shown as means ± sem. $^{\#\#\#}P < 0.001$, 2i-treated cells vs DMSO-treated cells; $^{***}P < 0.001$, RDF-treated cells vs DMSO-treated cells.

Supplementary Figure S3. RDF does not affect haESC apoptosis and the cell cycle of diploid ESCs

(A) Representative flow cytometry plots of apoptotic cell percentage in AG-haESCs

(AGH-OG-3) treated with DMSO or RDF. HaESCs were stained with anti-Annexin V antibody and 7AAD, and analyzed by flow cytometry.

(B) Relative fold changes of apoptotic cell percentage in AG (AGH-OG-3; HG165) and PG (319) -haESCs treated as in (A). Apoptotic cells were referred to Annexin V-positive cells. The results were compared RDF-treated haESCs to DMSO-treated haESCs.

(C) Representative flow cytometry pictures of cell-cycle distribution of diploid ESCs (E14) treated by DMSO or RDF.

(D) Relative fold changes of G0\G1-phase cell percentage in normal diploid ESCs (E14) and haESCs-derived diploid ESCs. The 4N cells purified from AG-haESCs (AGH-OG-3; HG165) and E14 cells were treated with DMSO or RDF, and analyzed by flow cytometry after Brdu antibody and PI staining.

(E) Relative fold changes of S-phase cell percentage in E14 and haESCs-derived diploid ESCs treated as in (D).

(F) Relative fold changes of G2\M-phase cell percentage in E14 and haESCs-derived diploid ESCs treated as in (D). Data are shown as means ± sem, *P < 0.05, **P < 0.01, ***P < 0.001.

Supplementary Figure S4. RDF modulates cell cycle gene expressions and promoter DNA methylation of *Cdc6* and *FGF5* genes

(A, B, C) Q-PCR analyses of cell-cycle regulator genes in AG (AGH-OG-3; HG165) and PG (319)-haESCs treated with DMSO or RDF in ES medium. A: AGH-OG-3 line;

B: HG165 line; C: 319 line. Data are shown as means ± sem. $^{*}P < 0.05$, $^{**}P < 0.01$, $^{***}P < 0.001$, RDF-treated cells vs DMSO-treated cells.

(D, E) Q-PCR analysis of cell cycle genes in AG-haESCs treated with DMSO, 2i, or RDF. D: AGH-OG-3 line; E: HG165 line. Data are shown as means ± sem. $^{*}P < 0.05$, $^{***}P < 0.001$, RDF-treated cells vs DMSO-treated cells.

(F) DNA methylation analysis of the promoter regions of cell-cycle regulator gene *Cdc6* and primed pluripotent gene *Fgf5* in AG-haESCs (AGH-OG-3) treated with DMSO or RDF in ES medium. The percentages of methylated CpG in total CpG were shown. Data are shown as means ± sem. $^{***}P < 0.001$, RDF-treated cells vs DMSO-treated cells.

Supplementary Figure S5. Wee1 kinase does not mediate the inhibition effect of RDF on self-diploidization.

(A, D and G) Flow cytometry analyses of the percentage of 1N cells in AG (HG165; AGH-OG-3) and PG (319) -haESCs treated with DMSO, RDF alone, or 300 nM PD166285 in the absence or presence of RDF in ES medium. A: HG165 line; D: AGH-OG-3 line; G: 319 line. Data are shown as means ± sem. $^{\#}P < 0.05$, $^{\#\#}P < 0.01$, RDF/PD166285-treated cells vs DMSO-treated cells at the same day; $^{*}P < 0.05$, $^{**}P < 0.01$, $^{***}P < 0.001$, RDF/PD166285-treated cells vs DMSO-treated cells at the same day.

(B, E and H) Q-PCR analyses of *Wee1 kinase* and *Fgfr1* genes in AG- and PG-haESCs treated with DMSO or RDF. B: HG165 line; E: AGH-OG-3 line; H: 319

line. Data are shown as means ± sem. $^{*}P < 0.05$, $^{**}P < 0.01$, $^{***}P < 0.001$, RDF-treated cells vs DMSO-treated cells.

(C, F and I) Q-PCR analyses of Wee1 upstream genes in AG and PG-haESCs treated with DMSO or RDF. C: HG165 line; F: AGH-OG-3 line; I: 319 line. Data are shown as means ± sem. $^{*}P < 0.05$, $^{***}P < 0.001$, RDF-treated cells vs DMSO-treated cells.

(J) Western blot analysis of Wee1 kinase expression. PG-haESCs (319) were cultured in ES medium added with DMSO or RDF. Beta-actin was used as the loading control.

Supplementary Figure S6. RDF/PD166285/2i promotes the proliferation of haESCs.

(A-C) Growth rate analyses of PG (319) and AG (HG165; AGH-OG-3)-haESCs cultured in ES medium added with DMSO, 2i, RDF/2i, PD166285/2i, or RDF/PD166285/2i. A: 319 line; B: HG165 line; C: AGH-OG-3 line. Data are shown as means ± sem. $^{*}P < 0.05$, $^{**}P < 0.01$, $^{***}P < 0.001$, Chemicals-treated cells vs DMSO-treated cells at the same time point.

(D) Flow cytometry analyses of 1N cell percentage in PG-haESCs (319). HaESCs were treated with DMSO, RDF, 300 nM PD166285, or the combination of RDF and PD166285 in 2i-ES medium. $^{\#\#\#}P < 0.001$, PD166285/2i-treated cells vs 2i-treated cells at the same day; $^{***}P < 0.001$, RDF/PD166285/2i or RDF/2i-treated cells vs 2i-treated cells at the same day.

(E) Representative pictures of karyotype analyses. AG-haESCs (AGH-OG-3) were treated with 2i, PD166285/2i, or RDF/PD166285/2i for 20 days. Red and blue frames

were used to mark representative haploid and diploid karyotypes, respectively.

(F) Q-PCR analyses of cell cycle genes in AG-haESCs (HG165) treated with 2i, or RDF/PD166285/2i. Data are shown as means ± sem. *P < 0.05, **P < 0.01, RDF/PD166285/2i-treated cells vs 2i-treated cells.

(G) Representative images of the chimeric embryos. RFP-marked haploid cells were cultured in 2i/PD166285/RDF condition for 15 days, and then injected into diploid embryos. Left: Brightfield; Middle: Fluorescence; Right: Merge. Data are shown as means ± sem, *P < 0.05, **P < 0.01, ***P < 0.001.

Supplementary Figure S1

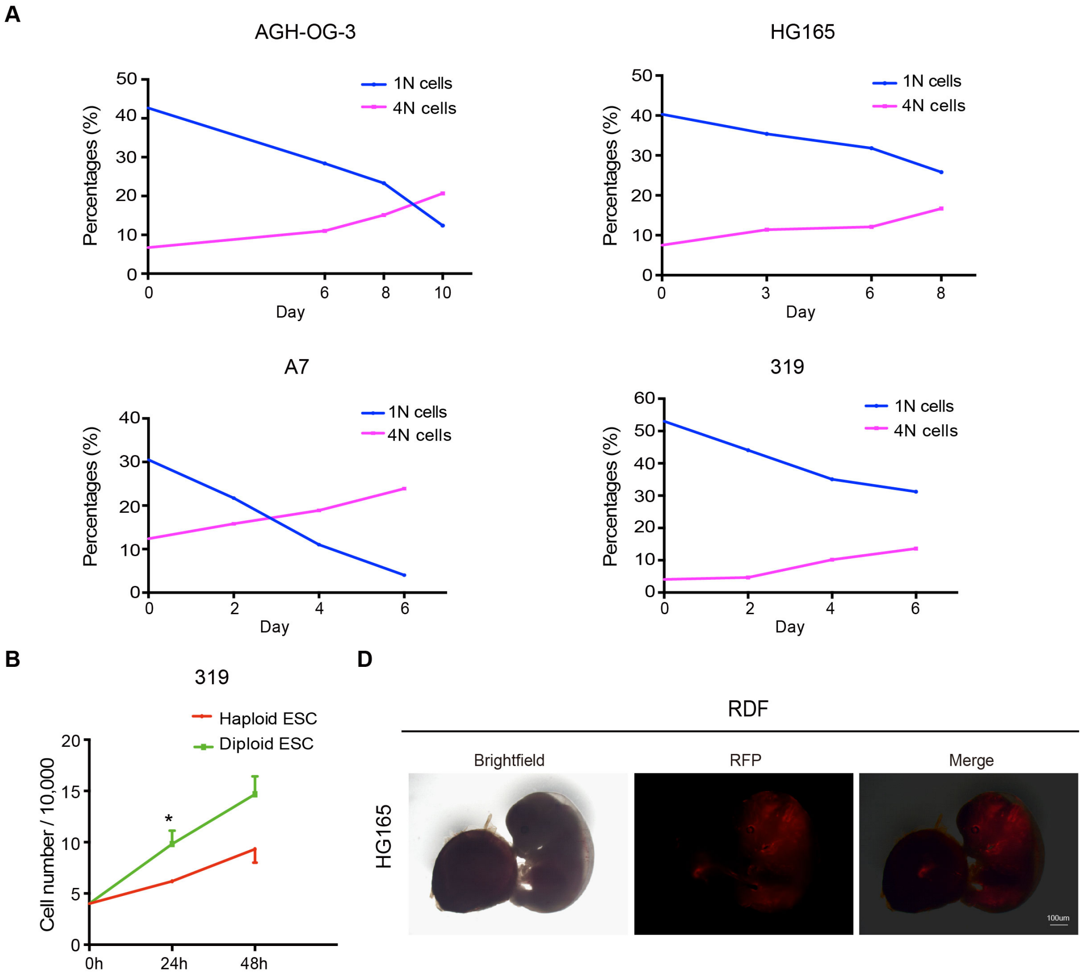

C

Name	Mechanism	Promote or inhibit self-diploidization
Pifithrin-α	P53 inhibitor	no
SB431542	ALK4, ALK5 and ALK7 inhibitor	no
Repsox	TGF-beta inhibitor	no
Cyclopamine	Hedgehog/SMO inhibitor	no
Su5402	FGF receptor inhibitor	no
Axitinib	VEGF receptor inhibitor	no
Dorsomorphin	ALK-2, ALK-3, and ALK-6 inhibitor	no
DMH1	BMP receptor inhibitor	no
Valproic acid	Histone deacetylase inhibitor	no
Chir99021	GSK3 inhibitor	no
Forskolin	Adenylate cyclase activator	no
XXI, Compound E	Y-secretase inhibitor	no

Supplementary Figure S2

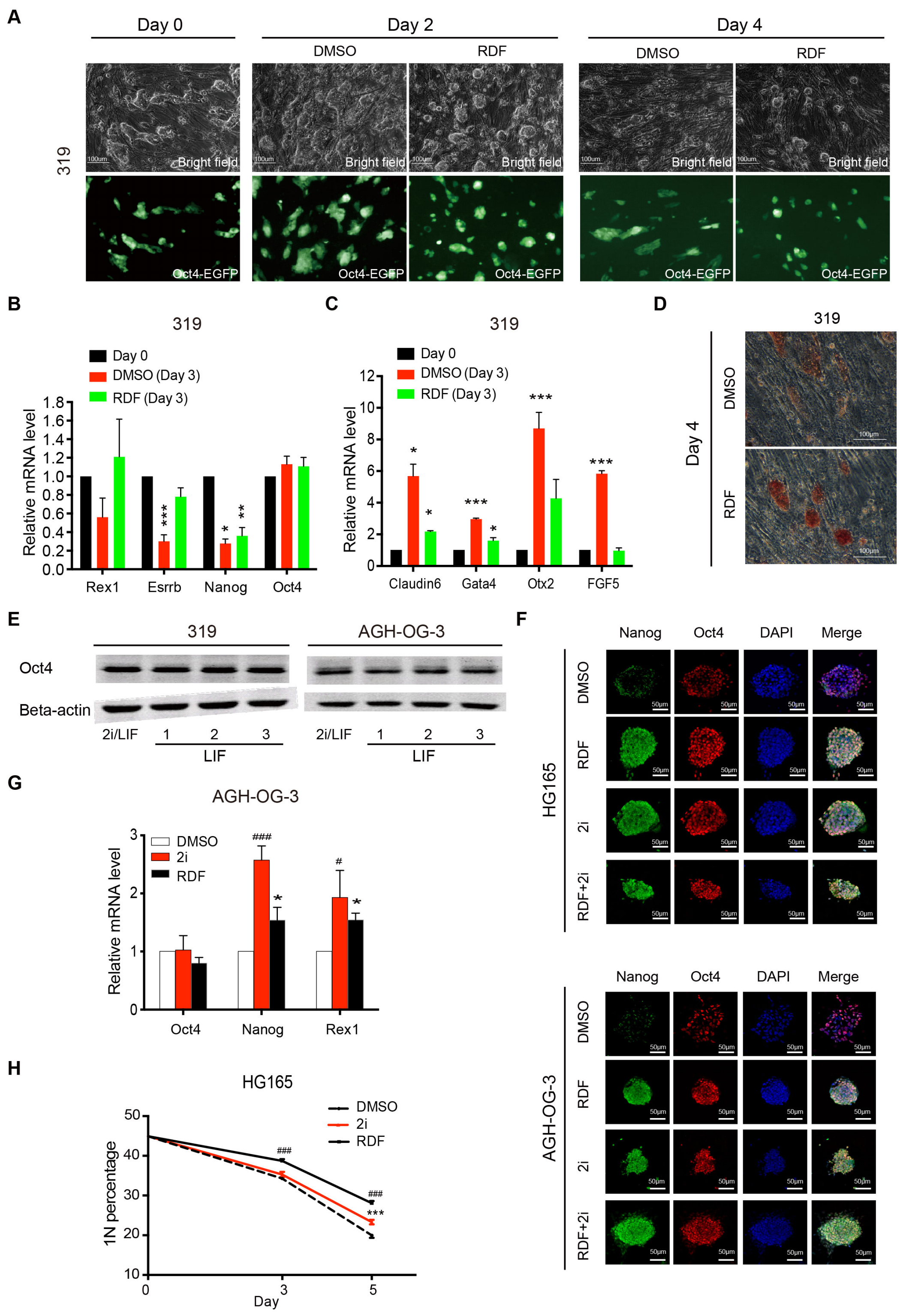

Supplementary Figure S3

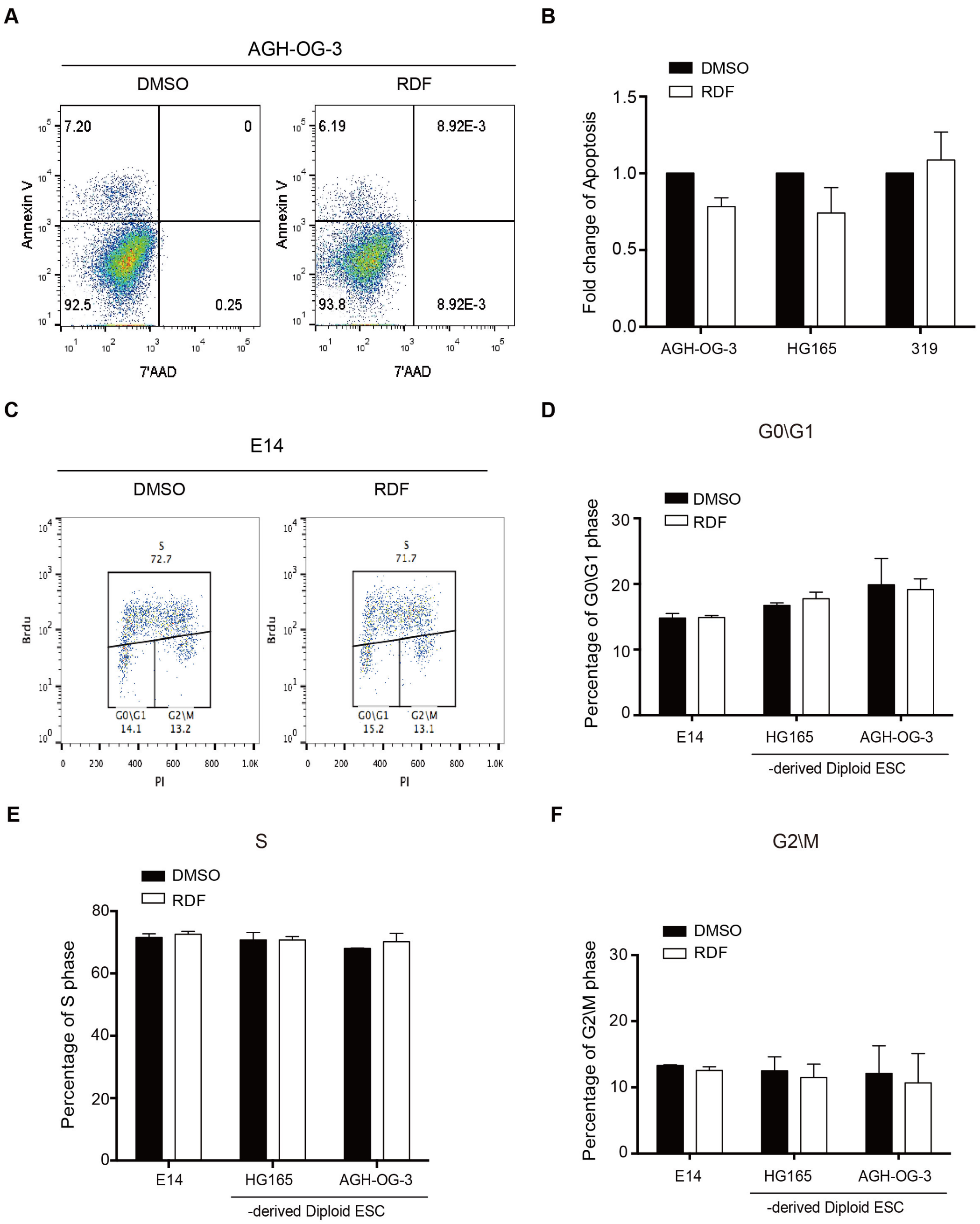

Supplementary Figure S4

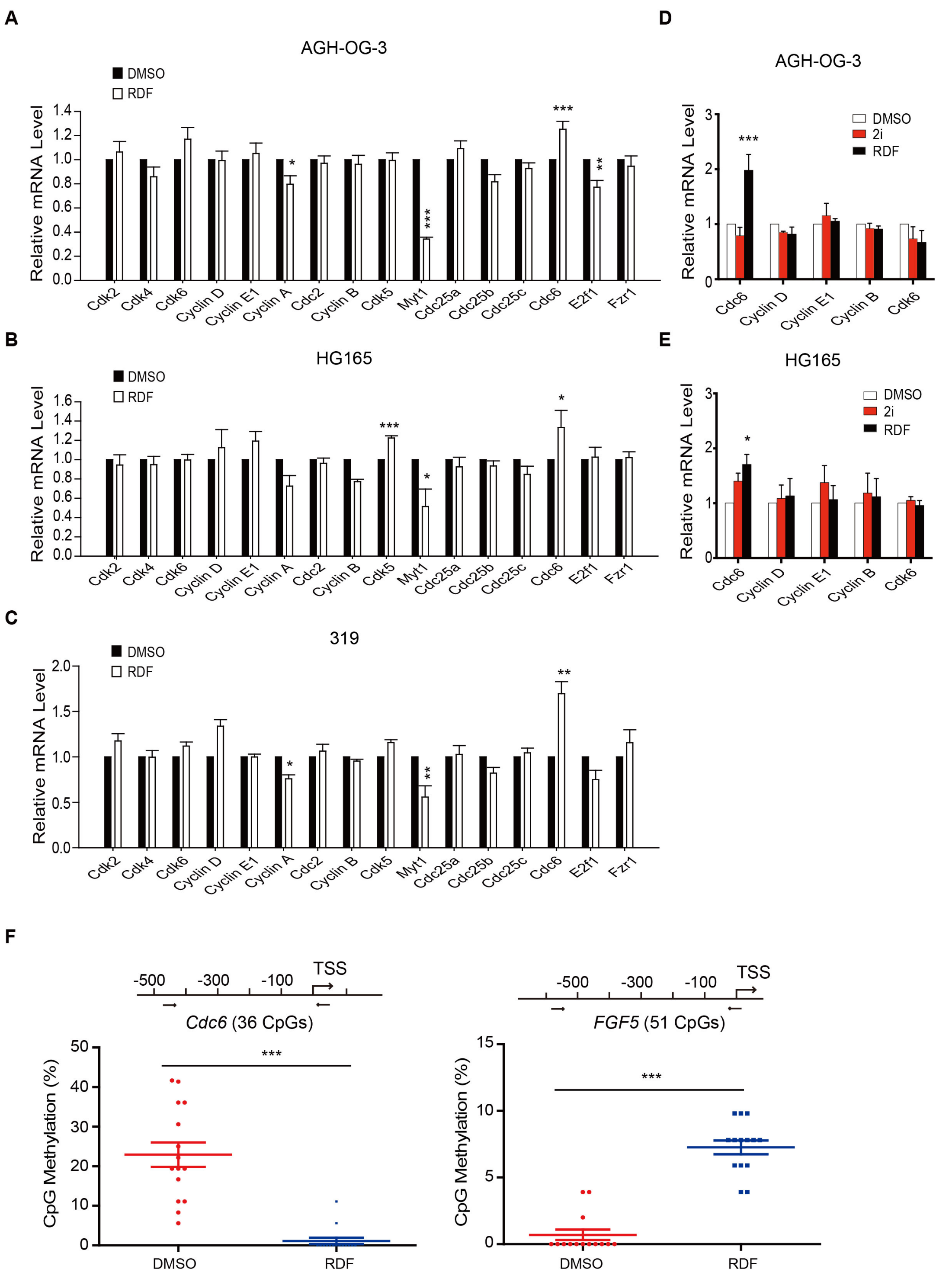

Supplementary Figure S5

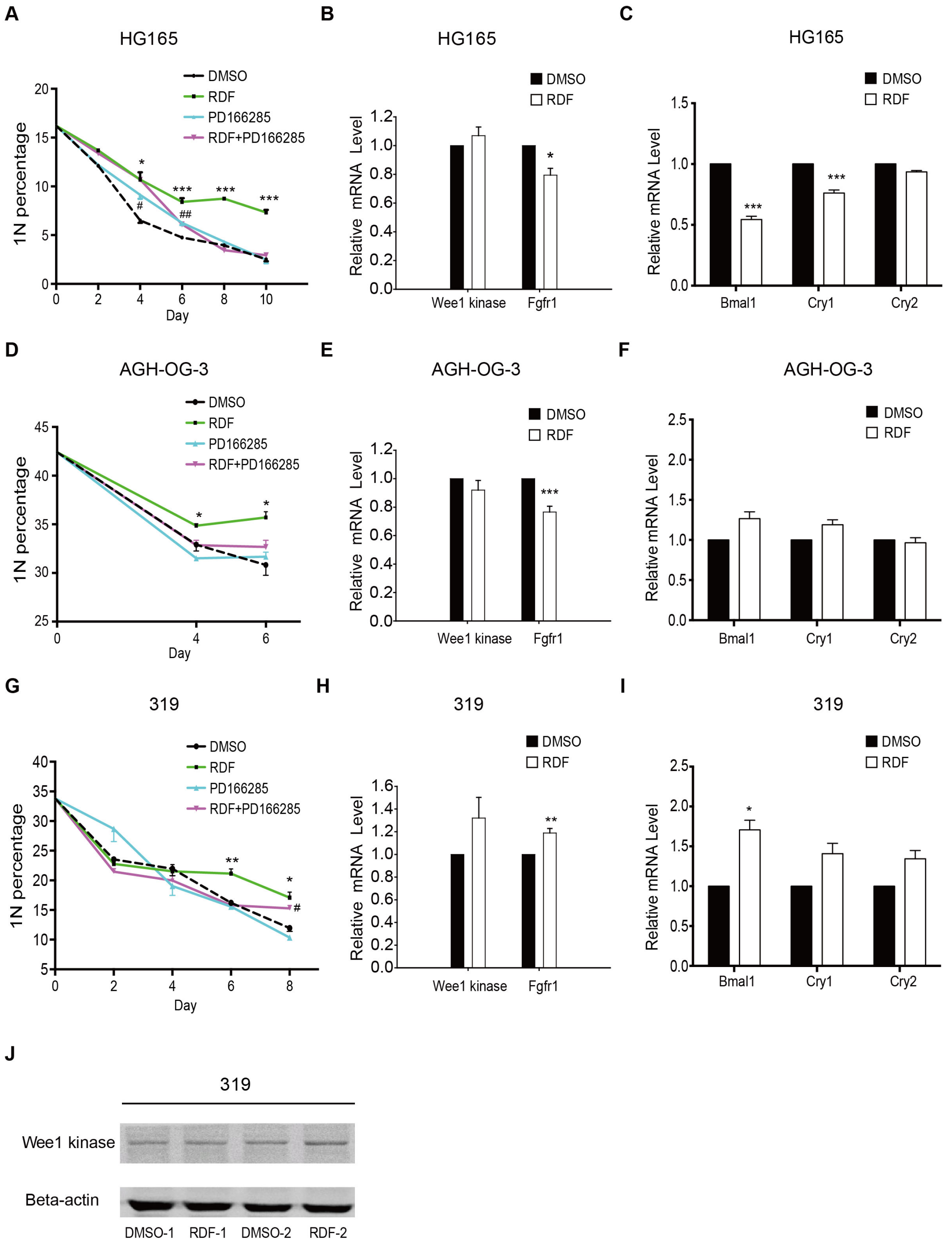

Supplementary Figure S6

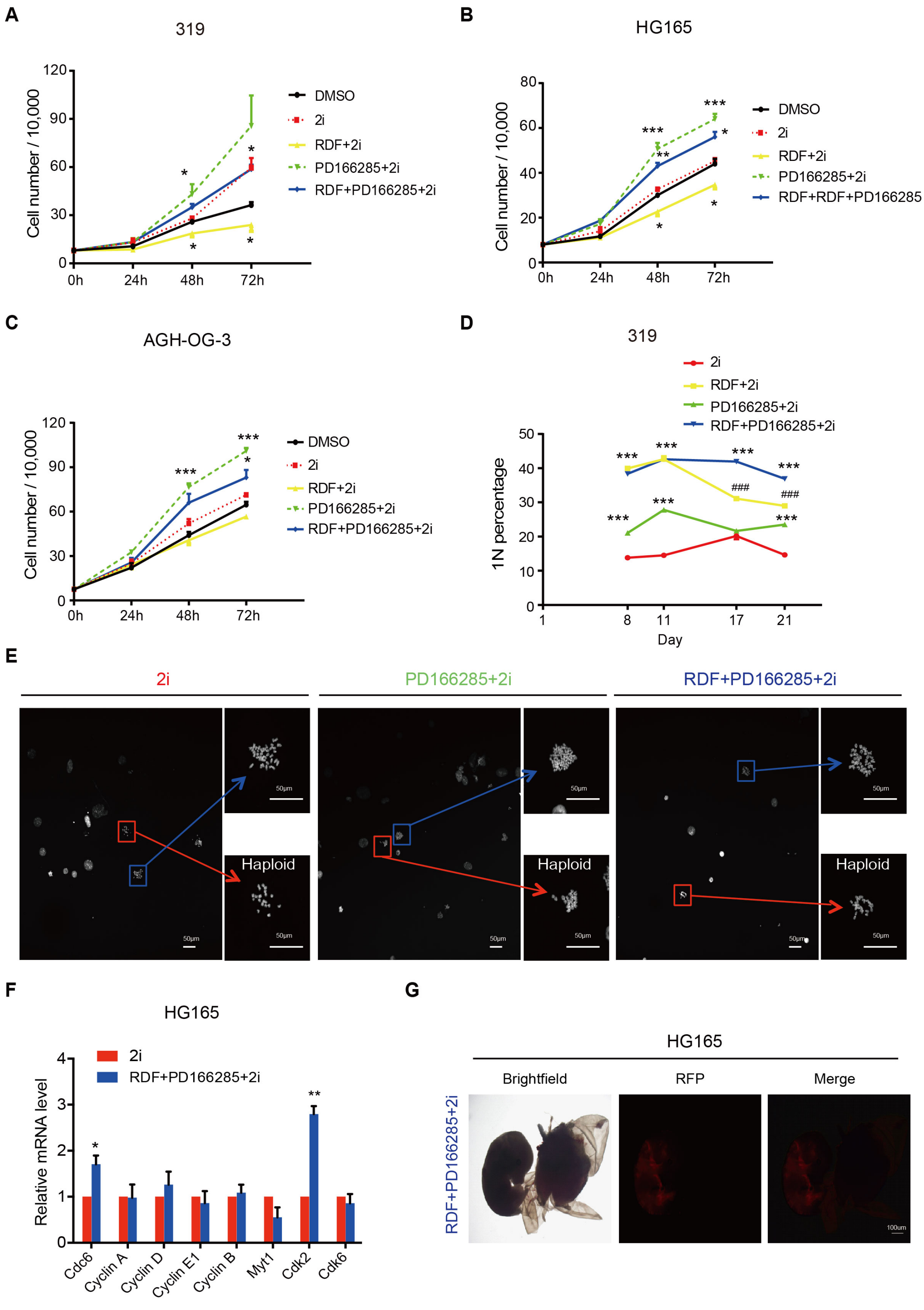

Supplement: Supplementary file 1 — Supplementary information [file 41598_2017_13471_MOESM1_ESM.pdf]
